# Sarcopenic Obesity Burden, Determinants, and Association With Risk of Frailty, Falls, and Functional Impairment in Older Adults With Diabetes: A Propensity Score Matching Analysis

**DOI:** 10.7759/cureus.49601

**Published:** 2023-11-28

**Authors:** Vansh Maheshwari, Saurav Basu

**Affiliations:** 1 Indian Institute of Public Health - Delhi, Public Health Foundation of India, New Delhi, IND

**Keywords:** older adults, functional impairment, falls, frailty, diabetes, sarcopenic obesity

## Abstract

Background: Sarcopenic obesity (SO) is a medical and functional state characterized by the coexistence of excessive adipose tissue and sarcopenia inside an individual. Recent epidemiological studies suggest a rising prevalence of SO in patients with diabetes mellitus (DM) probably due to the conducive environment resulting from adipose tissue dysfunction and muscle wasting accelerated by insulin resistance, chronic inflammation, and altered protein metabolism. Individuals with SO may have higher risk of experiencing falls, frailty, and disabilities due to compromised musculoskeletal integrity, gait abnormalities, and reduced functional capacity. The primary study objective was to determine the prevalence and predictors of SO among older adults with a history of DM. The secondary objective was to assess the association of falls, frailty, and functional disability with SO in patients with DM.

Methodology: This cross-sectional study analyzed data from the nationally representative Longitudinal Aging Study in India (LASI) Wave 1, focusing on individuals aged 60 and above, with an emphasis on diabetes. SO was assessed using the Asian Working Group for Sarcopenia criteria. Propensity score matching (PSM), logistic regression, and mediation analysis were used to explore relationships between diabetes, SO, and health outcomes (falls, frailty, and disabilities).

Results: Among 31,902 participants aged 60 and above, 14.26% had diabetes, and 17.67% of those with diabetes exhibited SO. Female gender (aOR = 2.63) and urban residence (aOR = 1.40) were significantly associated with higher odds of SO in individuals with diabetes. PSM analysis revealed a 9.0% higher likelihood of SO in older adults with DM than those without DM. SO was further linked to increased risk of falls, frailty, and various levels of activities of daily living (ADL) and instrumental activities of daily living (IADL) disabilities, with significant mediation effects ranging from 3.67% to 45.81%.

Conclusions: Diabetes substantially increases the risk of SO and associated functional disability with the risk of falls in older adults. Standard diabetes care should integrate health promotion especially nutrition to mitigate the risk of SO-linked functional disability and falls.

## Introduction

An estimated 536.6 million people worldwide have diabetes mellitus (DM), with the highest prevalence among those aged 40 to 57 years, with projections indicating a substantial increase, reaching 783.2 million (~12.2% of the global population) by 2045 [1]. The diabetes pandemic is predominantly driven by the syndemic of obesity with global trends indicating that 39% of adults are overweight and 13% are obese [2]. Sarcopenic obesity (SO) is a medical and functional state distinguished by the coexistence of excessive adipose tissue and sarcopenia inside an individual and is expected to impact up to 200 million people by 2051 [3]. The public health challenge of SO is accentuated by risk factors including the aging population influencing hormonal milieu, mitochondrial function, and inflammatory status and lifestyle factors especially unhealthy diet and lack of exercise that are associated with the risk of disease progression [4,5].

Recent epidemiological studies suggest a rising prevalence of SO in patients with DM probably due to the conducive environment resulting from adipose tissue dysfunction and muscle wasting accelerated by insulin resistance, chronic inflammation, and altered protein metabolism [6,7]. A meta-analysis of global studies has estimated the prevalence of SO in patients with DM to be 27% [8].

Individuals with SO may have higher risk of experiencing falls [9], frailty [10], and disabilities [11] due to compromised musculoskeletal integrity, gait abnormalities, and reduced functional capacity [12,13]. Additionally, it contributes to a frail phenotype characterized by diminished strength, endurance, and resilience [10,14]. SO impairs the performance of both basic activities of daily living (ADL), such as bathing and dressing, and more complex instrumental activities of daily living (IADL), including meal preparation and financial management as the compromised musculoskeletal function limits autonomy and independence in daily life [15].

India has the highest global burden of DM with increasing prevalence of overweight and obesity linked to an ongoing demographic, social, and nutritional transition [16]. Previous evidence has underscored the deleterious impact of both diabetes and SO on individual health. However, there remains a critical need to explore the intricate relationships among these conditions, particularly in the context of the Indian elderly population.

The primary study objective was to determine the prevalence and predictors of SO among older adults with history of DM. The secondary objective was to assess the association of falls, frailty, and functional disability (ADL/IADL) with SO in patients with DM. 

## Materials and methods

Study design and participants

This study utilized a cross-sectional design and included participants from the Longitudinal Aging Study in India (LASI) Wave 1 (2017-2018) dataset. The LASI Wave 1 dataset is a nationally representative survey conducted in India, which collects data on various health-related factors. The LASI gathered information from more than 72,000 individuals aged 45 and above, along with their spouses (of any age), spanning various states and union territories in India. The collected data encompassed a wide range of topics, including demographics, household economic status, chronic health conditions, health conditions based on symptoms, functional health, mental health (cognition and depression), biomarkers, health insurance, healthcare utilization, family and social networks, welfare programs, work and employment, retirement, satisfaction, and life expectations. Data for the LASI were collected through face-to-face interviews by trained field investigators.

The sample was derived from a multistage stratified cluster sample design, incorporating three phases for rural regions and four phases for urban regions. This design provides a scientifically robust foundation, allowing for standardized methodologies that facilitate comparisons with similar research conducted globally. Comprehensive and detailed information on the sample design, survey instruments, fieldwork procedures, data collection, processing methodologies, and response rates are available elsewhere [17]. The survey included a total sample size of 73,396 individuals. Our analysis focuses on eligible individuals aged 60 years and above, amounting to 31,902 participants, among whom 4,934 had DM.

Outcome measures

Primary Outcome

The primary outcome of this study was the occurrence of sarcopenic obesity among the study participants. SO is characterized by the coexistence of sarcopenia and obesity, with obesity being defined by a body mass index (BMI) greater than 24.99 kg/m^2^ [18]. Sarcopenia in LASI was defined according to the Asian Working Group for Sarcopenia (AWGS) criteria [19], requiring two out of three parameters: muscle mass, strength, and physical function. Due to the lack of muscle mass assessment in the survey, "possible sarcopenia" was determined based on muscle strength and physical performance following AWGS-2019 guidelines.

LASI employed a Smedley’s Hand Dynamometer, to measure grip strength in kilograms. Health investigators gathered two readings of grip strength for both the dominant and non-dominant hands. The calculation of muscle mass involved determining the average hand-grip strength score (in kilograms) from two consecutive trials on the dominant hand, adjusted for gender and BMI. A cutoff point of less than 26 kg for men and less than 18 kg for women indicated inadequate muscle strength.

Further, participants were instructed to perform the 4-meter walking test twice. Individuals who could not walk (even with an aid such as a cane, walker, or leaning on a wheelchair), suffered from dizziness, and/or had swelling or pain in their knee or hip were excluded from this activity [17]. The assessment of physical function included measuring slowness by averaging the time (in seconds) taken to cover a 4m distance. Individuals with a speed less than 0.8 m/sec were categorized as having a slow gait, while those with an equal or higher speed were considered to have a normal speed.

Participants were classified as possibly having sarcopenia if they exhibited both low hand grip strength (indicative of reduced muscle strength) and a slow gait (indicative of impaired muscle function).

Secondary Outcomes

Secondary outcomes of interest included falls, frailty, ADL, and IADL. These outcomes were assessed using validated scales and questionnaires, which were administered during the face-to-face interviews.

Falls were assessed through the self-reported question “Have you fallen down in the last two years?” and categorized in binary as ‘Yes’ or ‘No’.

Frailty was derived from the questions based on a modified Fried frailty phenotype scale [20]. Exhaustion, unintentional weight loss, weak grip strength, low physical activity, and slow walking time were the five components included in the physical frailty phenotype [21].

The ADL scale, based on five indicators (bathing, dressing, mobility, feeding, and toileting), was divided into three categories: "severe ADL disability" for those unable to perform any of the five activities, "moderate ADL disability" for those unable to function in less than five activities, and "No ADL disability" for those capable of performing all five activities.

Similarly, the IADL scale, covering seven instrumental activities (preparing a hot meal (cooking and serving), shopping for groceries, making telephone calls, taking medications, doing work around the house or garden, managing money, such as paying bills and keeping track of expenses and getting around or finding an address in an unfamiliar place), was categorized into "severe IADL disability" for those unable to perform any of the seven activities, "moderate IADL disability" for those unable to function in less than seven activities, and "No IADL disability" for those capable of performing all seven activities.

Explanatory variables

Socio-demographic variables included age (60-74, and ≥75 years), sex (male and female), education (no education/less than primary, primary complete, secondary, higher, and graduate and above), marital status (never married, currently married, widowed/divorced/separated/deserted), work status (not working and currently working), place of residence (rural and urban), and monthly per capita consumption expenditure (MPCE) quintile (Poorest, Poorer, Middle, Richer and Richest). Other potential lifestyle and health-related variables consist of tobacco consumption (no and yes, based on every use of tobacco products) and alcohol use (no and yes, based on every consumption of alcoholic beverages). Body mass index (BMI) was according to the WHO Pan Asian classification system [18].

Statistical analysis

Descriptive statistics were used to summarize the demographic and clinical characteristics of the study participants. The burden of sarcopenic obesity among previous diabetics was calculated as the prevalence rate, along with its associated 95% confidence interval. Propensity score matching (PSM) was done to assess the impact of diabetes on those who have SO. Cases were individually matched to controls, with diabetes being selected as the treatment status and confounding variables as the baseline characteristics. The effect of diabetes on SO was computed while controlling the background characteristics and other biases (related to the assignment of subjects in the treatment and control group). Further, multivariable binary logistic regression analysis was conducted to check for the associations between sarcopenic obesity among individuals with DM and its determinants. Both unadjusted and adjusted odds ratios (OR) were reported with a 95% confidence interval (CI). We considered a P<0.05 as statistically significant. Models were checked for any outliers and multicollinearity. We accommodated the cluster-sampling design of the Longitudinal Aging Study in India (LASI) by consistently applying relevant sampling weights.

Finally, a mediation analysis was undertaken to explore the potential mediating role of sarcopenic obesity in the interplay between DM and falls, frailty, ADL, and IADL disabilities. The Karlson-Holm-Breen (KHB) method, a robust statistical approach [22], was employed to evaluate both the direct and indirect effects of diabetes on falls, frailty, ADL, and IADL disabilities. In this analysis, the logistic regression model decomposed the total effect of diabetes into the sum of direct and indirect effects. The direct effect signifies the relationship of diabetes with these outcomes, after controlling for BMI and other covariates. On the other hand, the indirect effect illuminates the mediation impact of sarcopenic obesity on the association with physical frailty. The mediated percentage, reflecting the proportion of the association explained by the mediator variable, was estimated and deemed significant only when both total and indirect effects were statistically significant. All data analyses were conducted using Stata version 15.1 (StataCorp LLC, College Station, USA).

Ethical considerations

The LASI obtained ethical approval from the Indian Council for Medical Research (ICMR), ensuring the privacy and confidentiality of the research participants. Written Informed consent was obtained from all the participants prior to their inclusion in the study.

De-identified datasets were made available by International Institute for Population Sciences (IIPS), which also approved this study proposal. Since the LASI Wave I dataset is an anonymous publicly available dataset with no identifiable information of the participants, no separate ethical approval is required for this secondary data analysis.

## Results

Participant characteristics

The study included 31902 participants with a mean (SD) age of 69.17 (7.53) years, 77.25% of whom were aged 60-74 years, and 52.54% were females. The weighted prevalence of DM was 14.26% (n = 4934, 95% CI: 13.14, 15.45). Furthermore, SO was observed in 17.67% (95% CI: 13.12, 23.37) of the individuals with DM. This estimate is significantly higher than the prevalence (6.41%, 95% CI: 5.84, 7.03) observed in individuals without DM. Table 1 reports the sample characteristics of older adults aged ≥60 years with DM. 

**Table 1:** Socio-demographic and lifestyle characteristics of the elderly population DM: Diabetes mellitus

Characteristics	Total sample (N = 31902) n (weighted %)	Having DM (n = 4934) n (weighted %)
Age (years)		
60-74	25022 (77.25)	3994 (81.52)
75 and above	6880 (22.75)	940 (18.48)
Sex		
Male	15340 (47.46)	2486 (48.38)
Female	16562 (52.54)	2448 (51.62)
Education		
No education or less than primary	3835 (26.30)	622 (18.15)
Primary complete	3813 (25.73)	747 (23.18)
Secondary	4640 (31.95)	1146 (37.18)
Higher	1105 (7.72)	345 (11.6)
Graduate and above	1318 (8.30)	401 (9.89)
Marital status		
Never married	312 (0.72)	32 (0.32)
Currently married	20212 (61.63)	3322 (63.83)
Widowed/Divorced/Separated/Deserted	11378 (37.65)	35.84 (35.84)
Work status		
Not working	13476 (58.19)	2380 (71.05)
Currently Working	9373 (41.81)	880 (28.95)
Place of residence		
Rural	21085 (70.55)	2156 (46.57)
Urban	10817 (29.45)	2778 (53.43)
MPCE quintile		
Poorest	6580 (21.70)	697 (15.12)
Poorer	6573 (21.71)	823 (15.8)
Middle	6502 (20.95)	967 (18.29)
Richer	6259 (19.19)	1126 (24.65)
Richest	5988 (16.45)	1321 (26.15)
BMI (kg/m^2^)		
Underweight (<18.5)	6552 (26.67)	311 (7.90)
Normal (18.5-22.9)	10937 (38.27)	1259 (28.49)
Overweight (23.0-24.9)	3970 (12.92)	839 (17.99)
Obese (≥25.0)	6942 (22.14)	1956 (45.62)
Tobacco consumption		
No	19387 (59.82)	3550 (72.58)
Yes	12251 (40.18)	1349 (27.42)
Alcohol consumption		
No	26217 (85.41)	4211 (87.72)
Yes	5427 (14.59)	693 (12.28)

Determinants of SO among older adults with DM

The association of socio-demographic and lifestyle characteristics with SO among the older adults with DM was analyzed using multivariable binary logistic regression. On unadjusted analysis, age, sex, work status, residence, tobacco, and alcohol consumption were found to be significantly associated with SO among individuals with DM. Upon adjusted analysis, female gender (aOR = 2.63, 95% CI: 1.90, 3.64) and urban residence (aOR = 1.40, 95% CI: 1.03, 1.92) had significantly higher odds of having SO, while those currently working were less likely to have SO (aOR = 0.62, 95% CI: 0.42, 0.92) (Table 2).

**Table 2:** Association of socio-demographic and lifestyle characteristics of sarcopenic obesity (SO) among older adults with DM (N = 4934) *P<0.05, **P<0.001, Goodness of fit, P = 0.10, DM: diabetes mellitus; OR: odds ratio

Characteristics	SO Absent (n = 4130) n (%)	SO Present (n = 804) n (%)	Unadjusted OR [95% CI]	Adjusted OR [95% CI]
Age (years)				
60-74	3330 (80.92)	664 (19.08)	Ref	Ref
75 and above	800 (88.57)	140 (11.43)	0.55 [0.33, 0.90] *	0.78 [0.52, 1.17]
Sex				
Male	2221 (91.23)	265 (8.773)	Ref	Ref
Female	1909 (74)	539 (26.00)	3.65 [2.19, 6.10] **	2.63 [1.90, 3.64] **
Education				
No education or less than primary	503 (77.28)	119 (22.72)	Ref	-
Primary complete	625 (83.1)	122 (16.9)	0.69 [0.31, 1.55]	
Secondary	976 (78.31)	170 (21.69)	0.94 [0.28, 3.14]	
Higher	300 (90.6)	45 (9.4)	0.35 [0.17, 0.75] *	
Graduate and above	339 (82.82)	62 (17.18)	0.71 [0.35, 1.41]	
Marital status				
Never married	27 (89.06)	5 (10.94)	0.36 [0.06, 2.22]	-
Currently married	2847 (86.64)	475 (13.36)	0.45 [0.22, 0.92] *	
Widowed/Divorced/Separated/Deserted	1256 (74.6)	324 (25.4)	Ref	
Work status				
Not working	2016 (85.89)	364 (14.11)	Ref	Ref
Currently Working	787 (91.66)	93 (8.336)	0.55 [0.38, 0.80] *	0.62 [0.42, 0.92] *
Place of residence				
Rural	1867 (87.84)	289 (12.16)	Ref	Ref
Urban	2263 (77.53)	515 (22.47)	2.09 [1.21, 3.64] *	1.40 [1.03, 1.92] *
MPCE quintile				
Poorest	594 (87.07)	103 (12.93)	Ref	-
Poorer	701 (86.26)	122 (13.74)	1.07 [0.70, 1.65]	
Middle	807 (85.81)	160 (14.19)	1.11 [0.73, 1.69]	
Richer	938 (84.59)	188 (15.41)	1.23 [0.69, 2.19]	
Richest	1090 (72.67)	231 (27.33)	2.53 [1.06, 6.03] *	
Tobacco consumption				
No	2905 (79.42)	645 (20.58)	Ref	Ref
Yes	1191 (89.61)	158 (10.39)	0.45 [0.27, 0.73] *	0.87 [0.61, 1.25]
Alcohol consumption				
No	3485 (80.99)	726 (19.01)	Ref	Ref
Yes	615 (90.93)	78 (9.068)	0.42 [0.25, 0.72] *	0.97 [0.62, 1.50]
Comorbidities				
None	934 (88.39)	110 (11.61)	Ref	-
HTN/Lipid comorbidity	1617 (82.82)	311 (17.18)	1.58 [0.79, 3.14]	
Multimorbid	1579 (79.17)	383 (20.83)	2.00 [0.82, 4.88]	

PSM analysis

**Figure 1:**
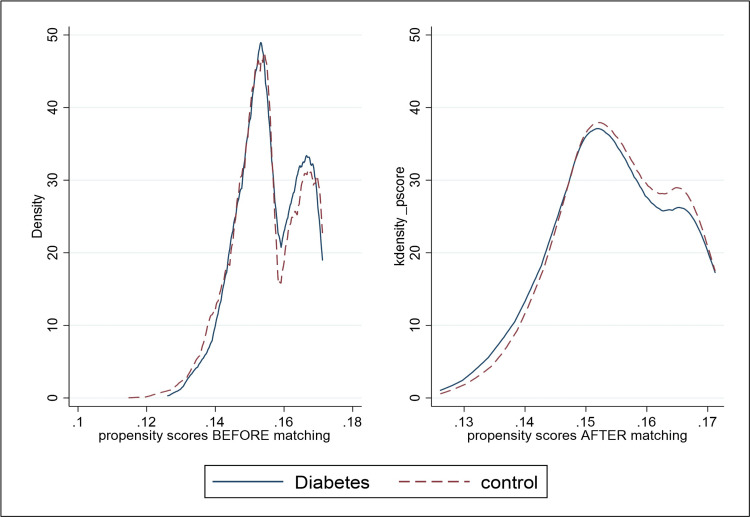
Balance plot for propensity score matching

The unmatched sample estimate, reflecting the raw estimate without matching was employed for comparison. The balance plot demonstrates the equilibrium of covariates between the treatment and control groups, both pre- and post-matching, affirming unbiased estimates of treatment effects (Figure 1).

Our analysis reveals a noteworthy finding: elderly adults with DM exhibited a 9.0% higher likelihood of having SO compared to those without DM. The average treatment effect (ATT) values, representing the difference in prevalence between treated and control groups, were 0.16 and 0.14, respectively. This suggests that, in the absence of DM, the prevalence of SO would have been lower among patients with DM. The average treatment effect on the untreated (ATU) values were 0.07 and 0.28 in the treated and control groups, respectively, implying that the development of diabetes in individuals without DM would increase their chances of experiencing SO by 21.0%. The overarching average treatment effect (ATE) stands at 0.18, indicating an 18.0% higher chance, on average, of having SO among patients with DM (Table 3).

**Table 3:** Propensity score matching of the effect of diabetes on sarcopenic obesity ATT: Average treatment effect on the treated; ATU: average treatment effect on the untreated; ATE: average treatment effect

Variable	Sample	Treated	Controls	Difference	Standard error (SE)	T-stat
Sarcopenic obesity	Unmatched	0.16	0.07	0.09	0.004	21.37
	ATT	0.16	0.14	0.03	0.048	0.56
	ATU	0.07	0.28	0.21	-	-
	ATE	-	-	0.18	-	-

Association of SO with the risk of frailty, falls, and functional impairment

The prevalence of falls and frailty was found to be 11.90% (95% CI: 8.85, 15.80) and 36.06 (95% CI: 24.61, 49.35), respectively in those having DM with SO comorbidity. The prevalence of moderate and severe ADL disabilities in the patients with DM was 19.90% (95% CI: 13.36, 28.59) and 1.87% (95% CI: 0.96, 3.59), respectively, while the prevalence of moderate and severe IADL disabilities was 60.12% (95% CI: 45.42, 73.20) and 1.97% (95% CI: 1.01, 3.82) respectively. To explore the potential mediating role of SO in the relationship between DM and falls, frailty, ADL, and IADL disabilities, a mediation analysis using the Karlson-Holm-Breen (KHB) method was conducted. The logistic regression model decomposed the total effect of DM into direct and indirect effects. The total effects of DM on falls, frailty, ADL, and IADL disabilities were indicative of significantly higher odds among those with DM. Indirect effect estimates for SO as mediator showed higher odds of having frailty (aOR = 1.07, 95% CI: 1.05, 1.09), ADL disability (aOR = 1.02, 95% CI: 1.01, 1.03), and IADL disability (aOR = 1.01, 95% CI: 1.003, 1.02) in those with DM. The analysis demonstrated a significant mediation effect of SO on the association between DM and falls, frailty, ADL, and IADL disability, respectively, with the mediated percentages of 3.67%, 45.81%, 5.92%, and 4.77%, respectively (Table 4).

**Table 4:** Mediated multivariable regression estimates of falls, frailty, ADL, and IADL disability ^a^Adjusted for sociodemographic and lifestyle characteristics, *P<0.05, **P<0.001, ADL: activities of daily living, IADL: instrumental activities of daily living, OR: odds ratio, CI: confidence interval

Mediation models	Falls Adjusted OR^a^ [95% CI]	Physical frailty Adjusted OR^a^ [95% CI]	ADL disability Adjusted OR^a^ [95% CI]	IADL disability Adjusted OR^a^ [95% CI]
Diabetes (key exposure)				
Total effect	1.29 [1.11, 1.51] *	1.15 [1.02, 1.30] *	1.29 [1.14, 1.46] **	1.31 [1.18, 1.45] **
Direct effect	1.28 [1.10, 1.50] *	1.08 [0.95, 1.22]	1.27 [1.13, 1.44] **	1.29 [1.16, 1.44] **
Indirect effect of sarcopenic obesity (mediator)	1.01 [1.00, 1.02]	1.07 [1.05, 1.09] **	1.02 [1.01, 1.03] *	1.01 [1.003, 1.02] *
Percent of effect mediated	3.67	45.81	5.92	4.77

## Discussion

The present study aimed to investigate the burden of SO among individuals with a history of DM and its association with various health outcomes. A notable 14.26% prevalence of DM was observed, aligning with the global trend of increased diabetes prevalence among the elderly [23]. Additionally, the prevalence of SO in individuals with a history of DM was 17.67%, emphasizing a substantial health burden associated with the concurrent presence of these conditions. However, the prevalence of SO in patients with DM observed in the present study is comparatively lower than that of a pooled estimate of global studies (27%) [8]. The comparatively lower prevalence of SO observed in this study apart from geographical variations may stem from variations in the selection criteria, heterogeneity in diagnostic standards, and variations in sample sizes when compared to previously reported studies.

The PSM analysis indicated a nearly one in five higher likelihood of SO in older adults with DM compared to those without DM, which is consistent with prior evidence. A biologically plausible interpretation is that the chronic inflammation and insulin resistance characteristics of DM may exacerbate the muscle loss and fat accumulation seen in SO [7,24]. Furthermore, the observation that female gender is a predictor of SO aligns with existing literature suggestive of gender-specific variations in body composition and muscle mass distribution attributable to hormonal influences, lifestyle factors, or societal norms affecting physical activity and dietary patterns [25].

In our study, urban residence was found as a significant predictor of SO. Urban environments often entail distinct lifestyle characteristics, including dietary habits, sedentary behaviors, and access to healthcare resources [26]. Understanding the interplay between urban living and the development of sarcopenic obesity is crucial for tailoring preventive strategies and interventions, especially given the global trend toward urbanization [27]. Conversely, current employment status was associated with lower odds of SO, suggesting a potential protective effect of regular physical activity associated with work [28].

In examining the downstream health implications of SO among individuals with DM, our analysis of falls, frailty, and disabilities in ADL and IADL is consistent with the previous evidence [29,30]. Mediation analysis revealed a substantial indirect effect of SO on the relationship between DM and frailty, ADL disability, and IADL disability.

The study's strengths include a large sample size, rigorous statistical methods, and consideration of confounding variables through PSM and multivariable regression analysis. However, limitations include the cross-sectional nature of the data, which hinders the establishment of causal relationships. Second, the study relies on self-reported data including that for diabetes, which may introduce recall bias and limit the generalizability of the study findings. Third, data on blood glucose levels were not available in the public domain leading to an underestimation of individuals with DM.

Our study carries important implications for public health interventions targeting older adults, particularly those with diabetes. Strategies aimed at preventing and managing SO may not only mitigate the risk of adverse health outcomes but also potentially alleviate the burden of DM-related complications. Hence, SO, exacerbated in the presence of diabetes, significantly heightens the risks of falls, and frailty, and compromises activities of daily living, reflecting the intricate interplay between musculoskeletal health and metabolic disorders in individuals with this complex comorbidity.

## Conclusions

Diabetes substantially increases the risk of SO and associated functional disability with the risk of falls in community-dwelling older adults in India. Individuals with diabetes have a significantly higher risk of developing SO as compared to those without diabetes, with about one in five of those with prior diabetes having SO. These findings emphasize the need for Standard diabetes care to integrate health promotion especially nutrition to mitigate the risk of SO-linked falls, frailty, and functional disabilities in daily living activities.

## Appendices

**Table 5:** STROBE Statement—checklist of items that should be included in reports of observational studies *Give information separately for cases and controls in case-control studies and, if applicable, for exposed and unexposed groups in cohort and cross-sectional studies. Note: An Explanation and Elaboration article discusses each checklist item and gives methodological background and published examples of transparent reporting. The STROBE checklist is best used in conjunction with this article (freely available on the Web sites of PLoS Medicine at http://www.plosmedicine.org/, Annals of Internal Medicine at http://www.annals.org/, and Epidemiology at http://www.epidem.com/). Information on the STROBE Initiative is available at www.strobe-statement.org

	Item No	Recommendation	Relevant section
Title and abstract	1	(a) Indicate the study’s design with a commonly used term in the title or the abstract	Title
		(b) Provide in the abstract an informative and balanced summary of what was done and what was found	Abstract
Introduction			
Background/rationale	2	Explain the scientific background and rationale for the investigation being reported	Introduction: 3^rd^ and 4^th^ paragraph
Objectives	3	State specific objectives, including any prespecified hypotheses	Introduction: 5^th^ paragraph
Methods			
Study design	4	Present key elements of study design early in the paper	Study design and participants: 1^st^ paragraph
Setting	5	Describe the setting, locations, and relevant dates, including periods of recruitment, exposure, follow-up, and data collection	Study design and participants: 1^st^ and 2^nd^ paragraph
Participants	6	(a) Cohort study—Give the eligibility criteria, and the sources and methods of selection of participants. Describe methods of follow-up Case-control study—Give the eligibility criteria, and the sources and methods of case ascertainment and control selection. Give the rationale for the choice of cases and controls Cross-sectional study—Give the eligibility criteria, and the sources and methods of selection of participants	Study design and participants: 2^nd^ paragraph
		(b) Cohort study—For matched studies, give matching criteria and number of exposed and unexposed Case-control study—For matched studies, give matching criteria and the number of controls per case	NA
Variables	7	Clearly define all outcomes, exposures, predictors, potential confounders, and effect modifiers. Give diagnostic criteria, if applicable	Outcome measures and Explanatory variables
Data sources/ measurement	8*	For each variable of interest, give sources of data and details of methods of assessment (measurement). Describe comparability of assessment methods if there is more than one group	Outcome measures and Explanatory variables
Bias	9	Describe any efforts to address potential sources of bias	Statistical analysis: 1^st^ paragraph
Study size	10	Explain how the study size was arrived at	Study design and participants: 2^nd^ paragraph
Quantitative variables	11	Explain how quantitative variables were handled in the analyses. If applicable, describe which groupings were chosen and why	Explanatory variables
Statistical methods	12	(a) Describe all statistical methods, including those used to control for confounding	Statistical analysis
		(b) Describe any methods used to examine subgroups and interactions	Statistical analysis
		(c) Explain how missing data were addressed	Statistical analysis
		(d) Cohort study—If applicable, explain how loss to follow-up was addressed Case-control study—If applicable, explain how matching of cases and controls was addressed Cross-sectional study—If applicable, describe analytical methods taking account of sampling strategy	Statistical analysis: 1^st^ paragraph
		(e) Describe any sensitivity analyses	
Results			
Participants	13*	(a) Report numbers of individuals at each stage of study—eg numbers potentially eligible, examined for eligibility, confirmed eligible, included in the study, completing follow-up, and analysed	Results: Participant characteristics
		(b) Give reasons for non-participation at each stage	NA
		(c) Consider use of a flow diagram	NA
Descriptive data	14*	(a) Give characteristics of study participants (eg demographic, clinical, social) and information on exposures and potential confounders	Results: Participant characteristics
		(b) Indicate number of participants with missing data for each variable of interest	Results: Participant characteristics
		(c) Cohort study—Summarise follow-up time (eg, average and total amount)	NA
Outcome data	15*	Cohort study—Report numbers of outcome events or summary measures over time	NA
		Case-control study—Report numbers in each exposure category, or summary measures of exposure	NA
		Cross-sectional study—Report numbers of outcome events or summary measures	Results: Participant characteristics and Determinants of SO among older adults with DM
Main results	16	(a) Give unadjusted estimates and, if applicable, confounder-adjusted estimates and their precision (eg, 95% confidence interval). Make clear which confounders were adjusted for and why they were included	Results
		(b) Report category boundaries when continuous variables were categorized	Results
		(c) If relevant, consider translating estimates of relative risk into absolute risk for a meaningful time period	Results
Other analyses	17	Report other analyses done—eg analyses of subgroups and interactions, and sensitivity analyses	Results: Propensity score matching analysis and Association of SO with risk of frailty, falls and functional impairment
Discussion			
Key results	18	Summarise key results with reference to study objectives	Discussion: 1^st^ paragraph
Limitations	19	Discuss limitations of the study, taking into account sources of potential bias or imprecision. Discuss both direction and magnitude of any potential bias	Discussion: 5^th^ paragraph
Interpretation	20	Give a cautious overall interpretation of results considering objectives, limitations, multiplicity of analyses, results from similar studies, and other relevant evidence	Discussion: 2^nd^, 3^rd^ and 6^th^ paragraphs
Generalisability	21	Discuss the generalisability (external validity) of the study results	Discussion: 6^th^ paragraph
Other information			
Funding	22	Give the source of funding and the role of the funders for the present study and, if applicable, for the original study on which the present article is based	Mentioned separately
